# Factors influencing the pace of food intake for nursing home residents with dementia: Resident characteristics, staff mealtime assistance and environmental stimulation

**DOI:** 10.1002/nop2.250

**Published:** 2019-03-06

**Authors:** Wen Liu, Ying‐Ling Jao, Kristine Williams

**Affiliations:** ^1^ College of Nursing The University of Iowa Iowa City Iowa; ^2^ College of Nursing Pennsylvania State University University Park Pennsylvania; ^3^ School of Nursing University of Kansas Kansas City Kansas

**Keywords:** dementia, eating performance, environmental stimulation, food intake, mealtime assistance, nursing home, older adult

## Abstract

**Aim:**

To examine the association of resident characteristics, staff mealtime assistance and environmental stimulation with the pace of food intake.

**Design:**

A secondary analysis of 36 baseline eating videos involving 19 nursing assistants and 15 residents with dementia in eight nursing homes from a communication intervention study.

**Methods:**

The outcome variable was the pace of food intake (the number of bites and drinks per minute). The exploratory variables were resident characteristics (age, gender, dementia stage and eating performance), staff mealtime assistance (frequency of verbal, visual, partial and full physical assistance) and environmental stimulation. Multi‐level models were used to examine the association.

**Results:**

A faster pace of food intake is associated with being male, better eating performance, staff provision of visual and physical assistance and better quality of environmental stimulation that involved interaction. The pace of food intake was not associated with resident age, staff verbal assistance or partial physical assistance.

## 1 INTRODUCTION

Dementia affects 5.5 million older adults (Alzheimer's Association, 2017) and 68–70% individuals living in the residential care settings (residents) in the United States (Thies & Bleiler, 2013). Residents with dementia commonly experience low food and fluid intake (Amella, 2002; Droogsma, van Asselt, & De Deyn, 2015; Liu, Cheon, & Thomas, 2014; Ullrich & McCutcheon, 2008). Prior research defines low food intake as the consumption of 75% or less of a meal and low fluid intake as <8 ounces of fluid consumed (Reed, Zimmerman, Sloane, Williams, & Boustani, 2005). Among residents with dementia, 31%–62% had low food intake and 46–63% had low fluid intake (Lin, Watson, & Wu, 2010; Reed et al., 2005).

Ensuring adequate food and fluid intake is an important component of dementia care with direct effects on hydration and maintenance of weight, which is one of the thirteen long stay nursing home quality measures (U.S. Centers for Medicare & Medicaid Services, 2017). In residents with dementia, inadequate food and fluid intake results in increased malnutrition and dehydration, which further lead to infection, weight loss, lower quality of life and increased morbidity and mortality (Hanson, Ersek, Lin, & Carey, 2013). Compared with cognitively intact residents, those with mild to moderate dementia experience a much higher chance of malnutrition and those with severe dementia are at a higher risk of dehydration (Guigoz, Jensen, Thomas, & Vellas, 2006).

## 2 BACKGROUND

The factors that influence food intake in dementia can be multi‐faceted. The Social Ecological Model provides a comprehensive framework for understanding the factors that may be associated with food intake by addressing the resident, caregiver, environmental and institutional levels (Bronfenbrenner, 1992).

### 2.1 Resident level

Residents with dementia experience cognitive and functional decline, behavioural and biological disturbances, taste alteration and smell dysfunction, loss of ability to tolerate the texture of regular diets, as well as comorbidities and medication side effects, which are almost universal and expected complications of progressive dementia (Droogsma et al., 2015). These complications further result in poor appetite, poor dentition, oral health‐related conditions, dysphagia and inability to plan meals and carry out complex eating tasks, which subsequently results in mealtime difficulties and further leads to insufficient food intake and weight loss (Droogsma et al., 2015; Liu, Shaw, & Chen, 2018). Specifically, 32%–87% residents with dementia demonstrate mealtime difficulties and require different levels of care (Chang, 2012; Lin et al., 2010; Liu et al., 2016). These individuals are often confused with food and the dining environment and demonstrate varied difficulties from choosing or locating food, preloading utensils and bringing food to the mouth, to chewing and swallowing food without pocketing, chocking or spitting food (Liu, Watson, & Lou, 2014). Mealtime difficulties are significantly associated with low food intake among residents (Keller et al., 2017; Lin et al., 2010; Liu, Williams, Batchelor‐Murphy, Perkhounkova, & Hein, 2019).

### 2.2 Caregiver level

Caregivers who assist residents with eating play an important role in engaging residents to ensure adequate food intake. Appropriate staff monitoring and assistance are associated with better food and fluid intake, while inadequate staff availability to assist and supervise residents contributes to low food intake (Abbott et al., 2013) and less likelihood of intake (Liu et al., 2019). Residents who receive physical help more often during mealtimes have more calories and protein intake, while those who receive physical help intermittently have less intake (Keller et al., 2017). However, previous reports have suggested that increasing staff time to assist with eating increased food intake by 11–15% in only half of the participants with low food intake (Simmons, Osterweil, & Schnelle, 2001).

The time spent assisting the resident may only partially explain the influence of staff assistance on food intake. The number and quality of caregiver‐resident (dyadic) interactions also matter since mealtime provides an opportunity for social interaction. Evidence from systematic reviews showed that insufficient evidence of mealtime assistance in supporting food intake, but mealtime assistance in combination with social interaction consistently improved eating independence, weight and quality of life (Abdelhamid et al., 2016; Liu, Watson et al., 2014). Adequate food and fluid intake was associated with the total number of interactions (Paquet et al., 2008), including both technical interactions such as verbal and non‐verbal prompts and social interactions such as commenting on food preferences and personal life experiences (Ullrich & McCutcheon, 2008). The quality of dyadic interaction as well as the caregiver's ability to support resident independence during mealtimes is positively associated with the amount of food intake (Amella, 2002). Altogether, it is important to understand how mealtime assistance and dyadic interaction during mealtimes influence residents' food and fluid intake.

### 2.3 Environmental and institutional level

The role of environmental factors is considered important to food intake but supporting evidence from the literature is inconsistent. Earlier studies found that improved dining environment characteristics in the residential care settings such as dining in public areas and having more non‐institutional features were significantly associated with increased intake for residents with dementia (Reed et al., 2005). A recent systematic review on the role of physical environment reported that well‐designed physical settings are crucial in creating a person‐centred dining environment to support best possible mealtime experience of residents (Chaudhury, Hung, & Badger, 2013). In contrast, a recent study found that the perceptions of the quality of the dining environment and the quality of the meal have very limited influence on intake (Buckinx, Morelle, & Bruyère, 2017). The finding is consistent with systematic reviews reporting that modification of dining environments or routines including tableware and table setting visual contrast (Brush, Meehan, & Calkins, 2002), noise reduction (McDaniel, Hunt, Hackes, & Pope, 2001), lighting enhancement (Brush et al., 2002; McDaniel et al., 2001), bulk food delivery service (Desai, Winter, Young, & Greenwood, 2007), family‐style meals (Altus, Engelman, & Mathews, 2002), smoothing or re‐creative music (Mc Hugh, 2012) have low level of evidence in improving meal intake (Abbott et al., 2013; Bunn et al., 2016; Liu, Watson et al., 2014). With the mixed results, the role of environmental factors on intake in dementia deserves further investigation.

### 2.4 Importance of the pace of food intake

Prior research on food intake mainly focuses on the amount of food and fluid consumption (Liu, Watson et al., 2014). Little research describes the pace of food and fluid intake or examines the factors that influence the pace of food and fluid intake in dementia. For residents with dementia, the pace of food intake, defined as the frequency of food and fluid intake within a designated time period, is directly related to the amount of intake. Given limited staffing and restricted meal time in residential care settings like nursing homes, residents with slow pace of food intake are more likely to have insufficient intake. Although overly rapid food intake may result in digestive issues such as dysphagia and chocking, most residents with dementia experience slow pace of food intake and ingestion due to cognitive decline and mealtime difficulties, putting them at higher risk for low food and fluid consumption during restricted meal time in residential care settings (Chang, 2012; Lin et al., 2010). Assessing the pace of food intake and examining the influencing factors are important to guide caregivers in providing dementia mealtime care more efficiently. Proper food intake pace helps ensure adequate food consumption and nutritional outcomes for residents with dementia (Chang, 2012; Lin et al., 2010).

This study aimed to (a) describe the pace of food intake among nursing home (NH) residents with dementia and (b) examine the association of the pace of food intake with resident characteristics, staff mealtime assistance and environmental stimulation. It was hypothesized that the pace of food intake would be significantly associated with resident characteristics (age, gender, dementia stage and eating performance), staff mealtime assistance (verbal, visual, partial physical and full physical assistance) and environmental stimulation.

## 3 DESIGN

This study was a secondary analysis of baseline videos collected from a dementia intervention study during 2011–2014 (Williams, Perkhounkova, Herman, & Bossen, 2016). The parent study was a randomized controlled trial that aimed to evaluate the efficacy of a nursing staff training programme to improve staff communication and decrease resistiveness to care among NH residents with dementia.

## 4 METHODS

### 4.1 Sample and setting

The parent study enrolled 127 staff and 83 residents from 13 NHs in Kansas. Residents were eligible if they had diagnosis of dementia, long stay status, resistiveness to care, capacity to hear staff communication and a surrogate decision‐maker available to provide informed consent. Staff were eligible if they were at least 18 years old, English speaking, a permanent NH employee and provided direct care for a participating resident at least twice a week over the previous month. A detailed description of the video recording procedures was reported elsewhere (Liu, Jao, & Williams, 2017; Williams et al., 2016).

For this study, a total of 505 baseline videos from the parent study were screened. Videos were selected for this analysis if they captured residents' eating and drinking activities at mealtimes regardless of the dining location. Videos were excluded if they only captured residents' taking medications no matter whether food was served or not, being transferred to or from the dining location, waiting for the meal to be served, sitting in front of the dining table with food being served but not eating or drinking. A detailed description of sample selection following exclusion and inclusion criteria was reported in another study using this video sample (Liu et al., 2017). The 36 eligible videos involved 15 residents with dementia and 19 nursing staff in 8 NHs. The duration of the videos varied from 18 s–10 min, depending on the length of the dyadic interaction. To maximize the sample size, this study included some short videos (7 videos ranging from 18–50 s) that captured adequate details for the coding of the pace of food intake, eating performance, staff mealtime assistance and environmental stimulation. The STROBE criteria for reporting observational studies were followed.

### 4.2 Data collection

Resident characteristics included age, gender, race, ethnicity and dementia stage. Dementia stage was determined by extracting data on Functional Assessment Staging in Alzheimer's Disease (FAST) from the Minimum Data Set (MDS) 3.0 (Sclan & Reisberg, 1992). The FAST score ranges from 1 (normal cognition and functioning) to 7 (severe dementia). Staff characteristics included age, gender, race, ethnicity, education, job title, number of years worked as a nursing caregiver and number of years worked in the study site. Residents' pace of food intake and eating performance, staff mealtime assistance and environmental stimulation were coded using computer‐assisted behavioural analysis of the videos.

*Pace of food intake *was conceptualized as the number of bites of solid food or drinks of fluids that a resident gets into the mouth and swallows per minute that were completed by the resident or facilitated by the staff. The total number of successful bites and drinks was counted by observing the video and then divided by the video duration in minutes to compute the pace of food intake for each resident. Coding of the pace of food intake and staff mealtime assistance demonstrated good inter‐rater reliability across two trained raters (*r* = 0.82–0.88).

*Eating performance *was measured by the adapted Level of Eating Independence (LEI) scale (Coyne & Hoskins, 1997). The scale consists of 9 items assessing the independence level in eating solids and drinking liquids. Each item was rated from 1 (total dependence)–4 (total independence), except the two swallowing items that are consistently scored as 4 as determined in the development of the LEI scale (Coyne & Hoskins, 1997). The total score ranges from 15–36, with a higher score suggesting better eating performance.

*Staff mealtime assistance *was conceptualized as whether staff provided verbal, visual, partial physical or full physical assistance to residents at mealtimes. The total number of each type of assistance provided was counted by observing the video following a coding protocol (Table 1). To account for the different lengths of the video sample, the total number of each type of assistance was divided by the video duration in minutes to compute the frequency of each type of assistance per minute. As the frequency of each type of assistance per minute was skewed towards less frequent, it was dichotomized such that the sample was divided into two groups: those who received assistance <1 time/min and those who received assistance ≥1 time/min. Coding of staff mealtime assistance demonstrated good inter‐rater reliability across two trained raters (*r* = 0.82–0.86).

**Table 1:** Coding protocol for four types of staff mealtime assistance

Assistance type	Coded one time whenever…
Verbal assistance	Staff provides verbal cues, prompts, positive reinforcement or encouragement to orient the resident to initiate, continue with or complete the meal
Visual assistance	Staff demonstrates role modelling of eating activities that the assisted resident can observe, or when staff provides visual cues to facilitate eating process (e.g., staff tapping table to show where the resident can put down utensils, finger point to the plate or cup indicating where the resident can pick up food or drinks)
Partial physical assistance	Staff preloads silverware with food, hands over food, hands over a container with a drink or utensils with food into the resident hand (e.g., handing over finger foods like bread or utensils like cups/forks with food and put into the resident's hand), or provides hand‐over‐hand or hand‐under‐hand feeding assistance to initiate or continue the meal
Full physical assistance	Staff provides complete feeding assistance and put food, drinks or utensils into the resident's mouth without involvement/input from resident

*Environmental Stimulation* was measured using the Person‐Environment Apathy Rating, Environment subscale (PEAR‐Environment), an observational scale designed to measure characteristics of environmental stimulation to capture physical, social and sensory features of the environment (Jao, Algase, Specht, & Williams, 2015). The stimulation can be any events, active objects, or people present that possibly trigger individuals' reactions, such as food, background music or a conversation that is present in the immediate environment where the resident is. The scale has six items evaluating clarity, strength and specificity of stimulation, as well as interaction involvement, physical accessibility and environmental feedback. Each item is scored from 1–4. Trained researchers conducted second‐by‐second coding for all videos using Noldus Observer^®^ XT10.5 software (Noldus Information Technology Inc., Leesburg, VA, USA) Then, a weighted average rating was calculated for each item in each video. For example, for a 5‐min video, if stimulation strength was rated as 2 for 1 min and 4 for 4 min, the weighted average rating of stimulation strength would be 3.6 (2*1/5 + 4*4/5 = 3.6). The weighted average ratings of the six items for each video were then summed up to represent the total score of environmental stimulation, which ranges from 6–24, with higher scores indicating more desirable environmental stimulation.

## 5 DATA ANALYSIS

Participant characteristics were analysed using descriptive statistics. Multi‐level linear modelling with maximum likelihood estimation was applied to estimate the role of resident characteristics, staff mealtime assistance and environmental stimulation characteristics on the pace of food intake using Stata 13.0 (StataCorp, College Station, TX, USA; Goldstein, 2003). A total of 18.1% of variance in the pace of food intake was accounted for by staff and resident‐level variations (intra‐class correlation coefficient = 0.1811, model 1), indicating a statistically significant clustering effect on the pace of food intake among observations within the same resident and among residents assisted by the same staff. Therefore, the clustering effects at resident and staff levels were adjusted for in all the models to demonstrate the independent effects of resident, staff and environment factors on the pace of food intake.

Exploratory variables included resident characteristics (age, gender, dementia stage and eating performance, model 2), staff mealtime assistance (verbal, visual, partial physical and full physical assistance, model 3), total score of environmental stimulation (model 4) and the six environment stimulation characteristics (model 5). The role of both overall environmental stimulation and individual environmental stimulation characteristics on the pace of food intake was of interest in this study. The correlation between the four types of staff mealtime assistance and six environmental stimulation characteristics was examined to avoid multicollinearity. There were very weak to moderate correlations (*r* = 0.10–0.52) between staff assistance and environmental stimulation, with statistically significant but weak to moderate correlations between verbal assistance and environmental feedback (*r* = 0.36, *p *= 0.029), verbal assistance and stimulation specificity (*r* = 0.34, *p *= 0.041) and partial physical assistance and stimulation specificity (*r* = 0.52, *p *= 0.001; Salkind, 2012). Thus, all four types of staff assistance and six environment stimulation characteristics were included in the model as they were not strongly correlated.

Coefficients with 95% C.I. for fixed effects of all covariates and the intercept were reported for each model. The log likelihood ratio of each model and the likelihood ratio difference were computed as appropriate (when the models used the same sample size) to compare the fit of the model to data. Assumptions were examined by the distribution of level‐1 residuals (i.e., histogram and Q‐Q plot). The level of significance was 0.05 for all the analyses.

## 6 ETHICS

The parent study and this study were approved by the Institutional Review Boards in the University of Iowa and University of Kansas. Written informed consent was obtained from both residents' family representatives and staff participants.

## 7 RESULTS

### 7.1 Sample characteristics

On average, residents were 86 years old (*SD* 8.29, range = 71–104) and had severe dementia based on FAST ratings (mean = 6.78, *SD* 0.17, range = 6.6–7). The residents were all white and predominantly non‐Hispanic (86.7%). Females represented 53% of the resident sample. The staff were on average 36 years old (*SD* 13.92, range = 71–104), worked for 11 years as clinical caregivers (*SD* 13.92, range = 1.5–31) and worked for almost 6 years in the study NH (*SD* 4.47, range = 0.2–13). They were predominantly female (89.5%), white (63.2%) and non‐Hispanic (84.2%). Thirty‐seven per cent of the sample were African American. All the staff were Certified Nursing Assistants (CNAs), with a few CNAs also working as activity assistants (5.3%) or in other aide roles (15.8%). Over half of them had at least some college education (68.4%).

Descriptive data for the pace of food intake, eating performance, environmental stimulation and staff mealtime assistance are shown in Tables 2 and 3. The 36 videos lasted for an average of 247 s with a range of 18–600 s. Food intake (bites of solids and drinks of fluids) occurred an average of 1.63 times/min, with a range from 0–3.6 times. Residents on average demonstrated a moderate level of eating performance and received a high level of environmental stimulation. Specifically, there was a moderate level of stimulation specificity, interaction involvement and environmental feedback with some variability. Stimulation clarity, stimulation strength and physical accessibility were rated consistently without any variability among all the videos. Both verbal and full physical assistance for ≥1 time/min were provided in less than half of the video recordings (41.7%). Partial physical assistance for ≥1 time/min was provided in only 8.0% of the videos and visual assistance for ≥1 time/min in only 11% of the videos.

**Table 2:** Characteristics of the pace of food intake, eating performance and environment stimulation

Variables (Measure)	Mean	*SD*	Range
Pace of food intake (number of bites and drinks per minute)	1.63	0.80	0–3.6
Eating performance (LEI)	27.08	5.16	19–36
Total video duration, s	247.44	203.87	18–600
Environment stimulation (PEAR‐Environment)	19.81	1.11	18–22.28
Stimulation specificity (to what extent the stimulation is delivered and tailored to the resident)	3.03	0.19	2.36–3.80
Interaction involvement (to what extent the stimulation includes interaction with the resident)	2.73	0.67	1.40–4
Environmental feedback (to what extent the stimulation prompts the resident to react)	3.04	0.45	2.18–3.90
Stimulation clarity (to what extent the stimulation is discernible and straightforward)	4.00	0	
Stimulation strength (to what extent the stimulation is substantial and unique)	4.00	0	
Physical accessibility (to what extent the stimulation is present and accessible without barriers for the resident)	4.00	0	

Note

The analysis included 36 eligible videos that involved 15 residents with dementia and 19 nursing staff in eight nursing homes.

**Table 3:** Characteristics of mealtime assistance provided by staff to residents

Frequency of assistance	*N*	%
Verbal assistance		
<1 time/min	21	58.3
≥1 time/min	15	41.7
Visual assistance		
<1 time/min	32	88.9
≥1 time/min	4	11.1
Partial physical assistance		
<1 time/min	33	91.7
≥1 time/min	3	8.3
Full physical assistance		
<1 time/min	21	58.3
≥1 time/min	15	41.7

Note

The analysis included 36 eligible videos that involved 15 residents with dementia and 19 nursing staff in eight nursing homes.

### 7.2 Factors influencing the pace of food intake

The association of resident characteristics, staff mealtime assistance and environmental stimulation with the pace of food intake is shown in Table 4. Model 3 that included both resident characteristics and staff mealtime assistance fit significantly better than model 2 (*χ* ^2^(*df*) = 37.08(4), *p *< 0.001). Model 4 that included overall environmental stimulation fit significantly better than model 3 (*χ* ^2^(*df*) = 8.05(1), *p *= 0.0046). Further, model 5 that included individual environmental stimulation characteristics fit slightly better than model 4 (χ^2^(*df*) = 6(2), *p *= 0.0497). Almost all of the 18% variance in the pace of food intake that was accounted for by the staff and resident clustering effects in model 1 was explained by the covariates in model 4 and model 5.

**Table 4:** The association of resident characteristics, staff mealtime assistance and environment stimulation with the pace of food intake using multi‐level linear modelling

Variables (measure or reference, range)	Model 1^a^	Model 2	Model 3	Model 4	Model 5
	Coefficient (95% CI)				
Resident age (years, 71–104)		−0.01 (−0.06, 0.04)	−0.01 (−0.04, 0.01)	−0.01 (−0.03, 0.02)	−0.01 (−0.04, 0.01)
Resident gender (0 = male, 0–1)		−0.54* (−1.06, −0.01)	−0.21 (−0.49, 0.07)	−0.28* (−0.53, −0.04)	−0.23* (−0.45, −0.01)
Dementia Stage (FAST, 6.6–7.0)		0.25 (−0.84, 1.35)	0.32 (−0.28, 0.93)	0.67* (0.10, 1.24)	0.59* (0.07, 1.11)
Eating performance (LEI, 19–36)		0.02 (−0.02, 0.07)	0.07*** (0.04, 0.10)	0.05** (0.02, 0.08)	0.06** (0.02, 0.09)
Verbal assistance (0 = <1 time/min, 0–1)			0.23 (−0.03, 0.50)	0.08 (−0.17, 0.33)	0.19 (−0.06, 0.46)
Visual assistance (0 = <1 time/min, 0–1)			0.93*** (0.46, 1.40)	0.84*** (0.44, 1.25)	0.93*** (0.48, 1.37)
Partial physical assistance (0 = <1 time/min, 0–1)			0.48* (0.01, 0.96)	0.26 (−0.16, 0.69)	0.40 (−0.11, 0.91)
Full physical assistance (0 = <1 time/min, 0–1)			1.00*** (0.69, 1.31)	0.83***(0.55, 1.12)	0.89***(0.62, 1.16)
Environment stimulation^b^ (PEAR‐Environment, 18–22.28)				0.20** (0.07, 0.33)	
Stimulation specificity (2.36–3.80)					−0.21 (−1.28, 0.85)
Interaction involvement (1.40–4)					0.48*** (0.23, 0.73)
Environmental feedback (2.18–3.90)					−0.24 (−0.63, 0.13)
Constant	1.57*** (1.29, 1.85)	1.04 (−11.09, 13.18)	−1.35 (−7.96, 5.24)	−7.94* (−15.01, −0.86)	−2.84 (−8.91, 3.21)
Log likelihood ratio	−42.21 (no *p* value)	−23.31	−4.77***	−0.75***	2.24***
Likelihood ratio difference, *χ* ^2^(*df*)			37.08(4)*** ^,^ c	8.05(1)** ^,^ d	6(2)* ^,^ e

Note

95% CI: 95% confidence interval; ICC: Intra‐class correlation coefficient.

The analysis included 36 eligible videos that involved 15 residents with dementia and 19 nursing staff in eight nursing homes.

a Model 1 only adjusted for the clustering effect at the staff and resident levels and did not include any variables or covariates. 18.11% of variance in food intake was accounted for at resident and staff levels (ICC = 0.1811). ICC was close to 0 once the resident‐level covariates were added in the model.

b The other three items of the PEAR‐Environment subscale were omitted from model 5 due to lack of variability.

c Comparison of model 3 and model 2.

d Comparison of model 4 and model 3.

e Comparison of model 5 and model 4. No comparison was available between models 1 and 2 due to different sample size in analysis.

*** *p *< 0.001,

** *p *< 0.01,

* *p *< 0.05.

The pace of food intake was higher among male residents (coefficient = 0.23, 95% CI = −0.45, −0.01) and residents with better eating performance (coefficient = 0.06, 95% CI = 0.02, 0.09). Resident dementia stage was significantly associated with the pace of food intake (coefficient = 0.59, 95% CI = 0.07, 1.11). However, the range of dementia stage in the study sample is very limited (FAST range = 6.6–7.0). The pace of food intake was higher among residents who were provided with visual assistance ≥1 time/min (coefficient = 0.93, 95% CI = 0.48, 1.37) or full physical assistance ≥1 time/min by caregivers (coefficient = 0.89, 95% CI = 0.62, 1.16). An eating environment with better stimulation quality was associated with more frequent intake of food (coefficient = 0.20, 95% CI = 0.07, 0.33). Particularly, food intake pace was associated with interaction involvement defined as the extent at which the stimulation includes interaction with the resident (coefficient = 0.48, 95% CI = 0.23, 0.73). Food intake pace was not significantly associated with resident age or the amount of verbal or partial physical assistance provided by staff.

## 8 DISCUSSION

This study examined the association of the pace of food intake with resident, staff and environmental characteristics in nursing home residents with dementia. The findings supported the hypothesis that the pace of food intake was associated with multiple resident characteristics (gender, eating performance), provision of visual assistance and full physical assistance by staff, the overall quality of environmental stimulation and specifically a dining environment that actively involved residents in the interaction.

### 8.1 Resident characteristics

The study found that being male and having a better eating performance were factors of higher pace of food intake. It is logical that residents who eat less frequently during the limited meal time period may have a lower intake. From this perspective, this finding was consistent with prior research reporting that female gender and eating dependence were associated with low intake amount (Lin et al., 2010) and that supporting resident independence in eating was associated with higher likelihood of intake (Liu et al., 2019). Although dementia stage was significantly associated with the pace of food intake, the finding should be interpreted with caution because the limited range of dementia stage in this sample may influence the estimates. It is possible that residents with severe dementia are more likely to receive more staff assistance and supervision, resulting in more frequent intake compared with those residents with less severe dementia. It is necessary to examine this relationship in a sample with a broader range of dementia stage.

As gender and dementia stage are less modifiable, improving eating performance, the most fundamental ADL among residents (Liu, Unick, Galik, & Resnick, 2015), has been a focus of recent intervention research. Montessori‐based activities and spaced retrieval trainings targeting older adults with dementia (Wu, Lin, Wu, Lin, & Liu, 2014) and staff mealtime assistance with a strong social interaction component (standardized verbal prompts, positive reinforcement, appropriate encouragement; Coyne & Hoskins, 1997; Van Ort & Phillips, 1995) have showed some evidence in decreasing feeding difficulty and improving eating performance (Abdelhamid et al., 2016; Bunn et al., 2016; Liu, Galik, Boltz, Nahm, & Resnick, 2015a, 2015b; Liu, Watson et al., 2014). Future work needs to test the impact of these strategies on the pace and amount food intake in dementia.

### 8.2 Caregiver mealtime assistance

This study found that the pace of food intake was significantly associated with visual and full physical assistance, but not with verbal and partial physical assistance. Specifically, residents provided with visual or full physical assistance at least once per minute showed faster food intake than those without. The study findings were consistent with prior research that showed staff provision of longer and continuous facilitation at mealtimes was associated with higher likelihood of intake (Liu et al., 2019). Findings from prior research on the association between staff assistance and the amount of food intake have been inconsistent in the dementia population. More recent studies have reported that full feeding assistance resulted in more oral intake and lack of feeding assistance was associated with low food intake in residents with severe dementia (Keller et al., 2017; Lin et al., 2010). Conversely, systematic reviews have reported that staff training programme on feeding skills (Chang & Lin, 2005), educational programmes (Suominen, Kivisto, & Pitkala, 2007) and feeding assistance interventions (Simmons et al., 2008) had insufficient evidence in supporting food intake in dementia (Abbott et al., 2013; Bunn et al., 2016; Liu, Watson et al., 2014). Moreover, different hand feeding techniques have shown inconsistent effects on food intake in dementia—direct hand feeding and under‐hand feeding techniques reduced eating difficulties and improved food intake compared with over hand feeding (Batchelor‐Murphy et al.., 2017). Findings of this study provide evidence to support the positive impact of using visual assistance and full physical assistance to improve residents' pace of food intake and thus potentially improve the amount of food and fluid consumption.

### 8.3 Environmental stimulation and interaction involvement

This study found that better quality of environmental stimulation was significantly associated with more frequent intake. Prior research reported inconsistent findings for the role of dining environment characteristics on food intake amount (Buckinx et al., 2017; Liu, Watson et al., 2014). Desirable and high‐quality environmental stimulation should be not only straightforward, substantial and physically accessible, but also individually tailored and directly delivered to the resident, interactively involving the resident and prompting the resident. Previously reported stimuli from caregivers that enhanced the overall quality of dining environment stimuli included providing menu picture cards for meal selection (Les Clarke, 2017), using finger foods (Soltesz & Dayton, 1995), providing liquid food when residents struggle with solid food (Liu et al., 2019), providing one food item at a time and using a flexible visual barrier (Cleary, 2009). As the dining environment stimuli include, but are not limited to, food, background music, conversations, noise, staff and other residents, it is important not to over‐stimulate residents so they stay focused on the primary stimulation—the meal. Less primary or unnecessary stimuli from the environment should be limited or carefully monitored to minimize distractions for optimal intake.

This study further found that an increased pace of food intake was associated with environmental stimulation that actively involved the resident during mealtimes (e.g., interpersonal conversation or non‐verbal interaction). This finding was consistent with prior work that examined the role of dyadic interaction quality on food intake amount in dementia (Amella, 2002; Ullrich & McCutcheon, 2008). Nursing staff, as direct care providers for individuals with dementia during mealtimes, have the most opportunities to provide straightforward, substantial and accessible stimuli to engage residents by appropriately modifying the dining environment and interacting with residents using verbal and non‐verbal strategies (Liu, Tripp‐Reimer, Williams, & Shaw, 2018). Training programmes that motivate nursing caregivers to interactively engage residents in mealtimes may increase pace and further improve the amount of food intake for residents with dementia. While most caregiver training programmes focus on the use of hand feeding skills^,^(Batchelor‐Murphy et al., 2017; Chang & Lin, 2005), training on engagement, motivation and dyadic interactions may promote interaction between caregivers and residents to further promotes food intake (Liu et al., 2015a, 2015b).

The association of food intake pace with environmental specificity and environmental feedback was not supported in this study. Prior research shows that environmental specificity is significantly associated with residents' eating performance (i.e., stimulations from the caregiver and dining environment that are directly tailored and individually delivered to individuals based on their needs and preferences are associated with better eating performance; Liu et al., 2017). With the findings that eating performance is associated with both stimulation specificity and the pace of food intake, future work may need to further examine the relationship between food intake pace and stimulation specificity, as well as the role of eating performance in this relationship in a more heterogeneous sample.

### 8.4 Implications for research

This study introduces the measure of the pace of food intake to assess the role of resident, caregiver and environmental characteristics on food consumption from an innovative perspective. The clinical usefulness of this measure is worthwhile to be further explored, especially in residential care settings. Future research needs to develop and use validated multiple‐item measures to better assess caregiver mealtime assistance and dyadic interaction and examine the association with intake outcomes. People with dementia may demonstrate high variability of eating behaviours and pace of food intake across different types of dementia or different meal types (e.g., breakfast, lunch and dinner). Future research need to examine the role of different types of dementia diagnosis and types of meals on the pace of food intake. Larger scale research using a heterogeneous sample with diverse dining environments is needed to examine the role of stimulation clarity, stimulation strength and physical accessibility. Lastly, the optimal pace of food intake may vary by resident depending on their overall cognitive and functional abilities during a particular meal. However, little is known about the recommended pace for food intake in older adults and future research is needed to accumulate evidence on the optimal pace of food intake and examine how the pace of food intake is associated with food consumption and nutritional status to establish clinical recommendations.

### 8.5 Implications for clinical practice

This is a pioneer study exploring the determinants of the pace of food intake. Better understanding of the factors that influence the pace of food intake can help promote person‐centred mealtime care for persons with dementia. Slow or prolonged chewing is a significant mealtime difficulty for persons with dementia (Liu, Watson et al., 2014). Caregivers may misinterpret this behaviour due to lack of knowledge or lack of patience and either improperly rush residents or stop prompting residents to conclude the mealtime prematurely. These task‐centred caregiving actions, including outpacing, verbally or physically controlling, ignoring or not interacting, are significantly associated with resistiveness to care and agitation in dementia (Gilmore‐Bykovskyi, Roberts, Bowers, & Brown, 2015). In contrast, adjusting to resident pace and including appropriate dyadic interactions are important components of person‐centred care and may reduce behavioural symptoms during mealtimes (Gilmore‐Bykovskyi et al., 2015). It is important to understand the pace of food intake and the factors that influence this outcome to promote person‐centredness of dementia mealtime care.

The results guide the development of novel interventions to address slow pace of food intake for nursing home residents with dementia. Providing high‐quality environmental stimulation along with appropriate assistance and interaction is essential to promote pace of food intake in this population. Multi‐level efforts targeting the resident, the caregiver, the food and dining environment, as well as the organizational context, are needed to ensure a successful mealtime experience (Liu et al., 2018) and have demonstrated feasibility as well as great potential to reduce feeding difficulty and improve food intake in residents with dementia and dysphagia (Chen et al., 2016). Clinical caregivers are critically positioned to provide optimal mealtime care for residents with dementia and caregiver–resident interaction affects food intake. Future clinical practice needs to incorporate pace of food intake as an important outcome in delivering multifactorial interventions in this population.

## 9 LIMITATIONS

The study included a small sample of eating videos. Dichotomous measures to assess the different types of caregiver assistance may limit the variability of these variables. Though dementia stage was controlled for in examining the factors that influence pace of food intake, the data on dementia types were not available and not controlled for. With lack of variation in dementia stage and some specific environmental stimulation characteristics, it was impossible to comprehensively examine the role of these characteristics. Additionally, as the pace of food intake was conceptualized as the number of bites or drinks that a resident gets per minute, the value for residents' pace of food intake in the videos that lasted <1 min in the study was primarily estimates of resident food intake pace patterns.

## 10 CONCLUSION

The findings provided preliminary information to support the association of resident characteristics, caregiver mealtime assistance and environmental stimulation with pace of food intake in residents with dementia. Insufficient food intake is a common and complicated problem, especially among frail residents at risk of weight loss and malnutrition, and requires multifactorial solutions. Eating performance, caregiver assistance and environmental stimulation, as modifiable factors to improve food intake, should be the focus of dementia mealtime care intervention research. Nursing staff need to assess residents' eating performance on a daily basis and engage residents in feeding themselves by providing appropriate assistance and interaction. Individualized and tailored mealtime care approaches are needed to overcome challenging mealtime scenarios and promote food intake for residents with dementia.

## CONFLICTS OF INTEREST

No conflict of interest has been declared by the author(s).

## AUTHOR CONTRIBUTIONS

All authors contributed to the study conceptualization, interpretation of findings, and manuscript preparation and revision. Wen Liu led video coding for eating performance, staff mealtime assistance and pace of food intake, and performed statistical analysis. Ying‐Ling Jao led video coding for environmental stimulation. Kristine Williams is the Principal Investigator (PI) of the parent study. All authors agreed to be accountable for all aspects of the work in ensuring that questions related to the accuracy or integrity of any part of the work are appropriately investigated and resolved.
